# New Drugs and Praparations

**Published:** 1894-04-21

**Authors:** 


### New Drugs and Preparations.

[We shall be glad to receive, at our Office, 428, Strand, London, W.C., from the manufacturers, specimens of all new preparations and drugs which may be brought out from time to time.]

### FERRATIN.

This is a preparation of iron first brought into notice by Schmiedeberg.[1] It has long been known that iron in the body always exists as a very complex organic molecule such, for example, as that of hæmoglobin. Bunge[2] was the first to prepare such a body; he obtained it from the yolk of egg; it contained 0·29 per cent. of iron, and he called it hæmatogen. Dr. Marfori, working under Schmiedeberg's directions, has prepared from the pig's liver one of these ferrated proteid compounds; it is much more highly ferrated than Bunges, for it contains 6 per cent. of iron, and he calls it ferratin. This he believes is the form in which iron exists in the food and the tissues, and that hence it is likely to be of use in supplying the organism with the necessary iron, which in ordinary circumstances should be furnished by the food, and that in this way ferratin may be useful in promoting growth and nutrition, or in removing morbid conditions. The first ferrated bodies prepared by Marfori[3] were too expensive and did not contain the right proportion of iron, but ferratin is not dear, and is of suitable strength of iron. As regards its physiological action, it is not capable of being replaced by other compounds of iron. It is particularly easy of assimilation, and never upsets digestion, but rather improves it. The dose should be such that some of the ferratin is sure to escape decomposition from the hydrochloric acid in the gastric juice; only sufficient is absorbed for the use of the body, therefore there is no fear of any harmful results. Ferratin is thus primarily a food, and is especially valuable in chlorosis, but to a less degree in any illnesses for which preparations of iron are usually recommended. It is a fine powder of a reddish-brown or rusty colour, and is prepared in two forms—one uncombined and insoluble in water, the other as a sodium compound which readily dissolves in water after being shaken up and allowed to stand for a while. The water used must, however, be free from calcium salts, as otherwise an insoluble calcium compound may be easily found. The watery solutions of sodium ferratin may with advantage, especially for young children, be added to milk or other liquid food. Both forms, however, the soluble and the insoluble, may be taken in simple powder without any vehicle. For children the daily dose is two to eight grains, for adults 15 to 25, divided into two or three doses. No special attention to diet is required, but it is better to avoid acidulous articles of food lest they should lead to the decomposition of some of the ferratin.

[1] Practitioner, Dec., 1893, and Archiv. f. Exper. Pathologie u. Pharmakologie, Bd. xxxiii. [2] Pathological and Physiological Chemistry. [3] Archiv. f. Exper. Pathologie u. Pharmakologie, Bd. xxix.

